# Clinical and Functional Outcomes of Peroneus Longus Versus Hamstring Tendon Autografts in Anterior Cruciate Ligament Reconstruction: A Prospective Interventional Comparative Study

**DOI:** 10.7759/cureus.104890

**Published:** 2026-03-09

**Authors:** Mohd Rafiuddin Warsi, Pavneesh Kumar, Vikrant Chauhan, Anil K Joshi, Akshat Mittal

**Affiliations:** 1 Orthopaedics, Government Doon Medical College and Hospital, Dehradun, IND

**Keywords:** acl reconstruction, faam (foot and ankle ability measure), functional outcome, hamstring autograft, ikdc scores, peroneus longus

## Abstract

Background: Selection of an optimal autograft for anterior cruciate ligament (ACL) reconstruction remains debatable due to concerns regarding graft adequacy and donor-site morbidity. This study compared the clinical and functional outcomes of peroneus longus tendon and hamstring tendon autografts in ACL reconstruction.

Methods: In this prospective comparative study, 60 patients underwent arthroscopic single-bundle ACL reconstruction for unilateral ACL tears using autografts of either hamstring (n = 30) or peroneus longus tendons (n = 30), followed by a standardized rehabilitation protocol. Knee function was assessed using the International Knee Documentation Committee (IKDC) score, and ankle function using the Foot and Ankle Ability Measure (FAAM) preoperatively and at one, three, and six months following the intervention.

Results: Baseline characteristics were comparable between groups. Graft length and tunnel dimensions were similar, while graft thickness was significantly more in the peroneus longus group (p < 0.001). Improvement in IKDC scores was significant in both groups; the hamstring group showed better scores at three months (p = 0.001), with no significant difference at six months. FAAM scores were comparable at all follow-ups, demonstrating preserved ankle function in the peroneus longus group.

Conclusion: Peroneus longus tendon autograft provides comparable short-term functional outcomes to hamstring tendon autograft, with the advantage of greater graft thickness and no clinically significant ankle morbidity.

## Introduction

Among knee ligament injuries, the anterior cruciate ligament (ACL) is most frequently affected, with a higher incidence seen in young, physically active individuals, particularly athletes, while ACL reconstruction is widely accepted as the treatment of choice for patients with symptomatic instability, aiming to restoration of knee stability, improve functional outcomes, and to facilitate restoration of daily activities and sports [1,2]. Despite advancements in surgical techniques and fracture fixation methods, an optimal autograft remains a subject of challenge for surgical choice [3].

Hamstring tendon autograft has been extensively used in ACL reconstruction because of its favorable biomechanical properties, ease of harvest, and reduced anterior knee pain compared with bone-patellar tendon-bone grafts [4,5]. However, concerns have been raised regarding hamstring weakness, graft size variability, and potential effects on dynamic knee stability, particularly in patients with smaller tendon dimensions [6,7]. Increased risk of graft failure has been found to be associated with inadequate size of the graft and graft diameter, prompting the need for alternative autograft sources that provide sufficient graft without compromising function [8].

The suitability of the peroneus longus tendon as an alternative graft for ACL reconstruction has been supported by anatomical and biomechanical studies demonstrating adequate graft length, diameter, and tensile strength required for effective ligament reconstruction [9,10]. Moreover, several biomechanical and clinical studies have reported that peroneus longus tendon autografts yield larger graft diameters as compared with hamstring tendons, which may be advantageous in reducing graft failure risk [11-13]. Nevertheless, concerns persist regarding possible donor-site morbidity, particularly the impact of tendon harvest on ankle stability and function [14].

Recent comparative studies have suggested that ACL reconstruction using the peroneus longus tendon results in knee functional outcomes comparable to those achieved with reconstruction using the hamstring tendon autograft, while preserving ankle functions [11,15-17]. However, variations in study design, follow-up duration, and outcome measures have limited the generalizability of these findings. In addition, data on the temporal pattern of knee and ankle functional recovery during early and mid-term follow-up remain limited.

Therefore, this prospective comparative study was designed to compare the post-surgery clinical and functional outcomes of peroneus longus tendon versus hamstring tendon autografts in patients undergoing ACL reconstruction following a knee injury. The International Knee Documentation Committee (IKDC) score was used to assess the knee function, while ankle- and foot-related function was evaluated using the Foot and Ankle Ability Measure (FAAM). By analyzing graft characteristics and functional outcomes over a six-month follow-up period, the present study seeks to further elucidate the long-term effectiveness of the peroneus longus tendon autograft as an alternative to the hamstring tendon autograft in anterior cruciate ligament reconstruction.

## Materials and methods

This prospective comparative study was conducted at the Department of Orthopedics, Government Doon Medical College and Associated Doon Hospital, Dehradun, Uttarakhand, after approval from the Institutional Ethics Committee from October 2023 to July 2025. Written informed consent was obtained from participants after explaining in vernacular about all the aspects of the surgical intervention and possible positive or negative outcomes. The participants were free to remain in the study or to leave as per their consent.

The sample size was calculated using a power analysis based on expected differences in IKDC scores between groups. Assuming a mean difference of five points, a standard deviation of 8, power of 80%, and α error of 0.05, the minimum required sample size was calculated to be 27 patients per group. Considering possible dropouts, 30 patients were enrolled in each group.

Sixty patients with clinically and MRI-confirmed unilateral anterior cruciate ligament (ACL) tears were enrolled, and patients were allocated into two groups using a computer-generated random number sequence: hamstring tendon autograft (n = 30) and peroneus longus tendon autograft (n = 30). Patients aged 18-45 years with symptomatic knee instability were included. Exclusion criteria comprised multi-ligament injuries, previous knee surgery, fractures around the knee, advanced osteoarthritis (Kellgren and Lawrence Grades 3 and 4), neuromuscular disorders, and pre-existing ankle pathologies.

All patients underwent ACL reconstruction using an arthroscopic single-bundle autograft technique performed by the same surgical team. In the hamstring autograft group, semitendinosus and gracilis tendons were harvested and prepared as a quintuple graft. In the peroneus longus group, the peroneus longus tendon was harvested proximally to the lateral malleolus, preserving the peroneus brevis tendon, and prepared as a triple graft. Graft diameter and length, as well as femoral and tibial tunnel lengths, were measured intraoperatively. Autograft fixation was achieved using standard fixation devices.

All patients followed a standardized rehabilitation protocol: non-weight-bearing with a brace and isometric quadriceps exercises for the first two weeks; from weeks two to four, initiation of range-of-motion (ROM) exercises and non-weight-bearing ambulation with a walker; from weeks 4-10, progression from partial to full weight-bearing with continued ROM and strengthening; from weeks 11-24, ongoing strengthening and ROM exercises; and from weeks 25-52, advanced strength and agility training, with return to sports or heavy labor once functional parameters were comparable to the contralateral limb.

The International Knee Documentation Committee (IKDC) subjective knee score was used to assess clinical outcomes, while ankle function was evaluated using the Foot and Ankle Ability Measure (FAAM) preoperatively and after one, three, and six months postoperatively [18,19]. Table 1 provides descriptions and interpretations of the functional outcome measures (IKDC and FAAM Scores).

**Table 1:** Description and interpretation of functional outcome measures (IKDC and FAAM scores). IKDC: International Knee Documentation Committee; FAAM: Foot and Ankle Ability Measure; ADL: activities of daily living. Scores for the IKDC and FAAM subscales are standardized to a 0–100 scale or percentage, with higher scores indicating better function and fewer symptoms. IKDC score categories: 0–40 = poor, 40–60 = fair, 60–80 = good, and >80 = excellent. FAAM scores are calculated as the percentage of the maximum possible score achieved. This table is independently created by authors using the information from the sources [18,19].

Outcome measure	Domain	Score range	Calculation method	Interpretation
International Knee Documentation Committee (IKDC) Subjective Knee Score	Knee symptoms, sports activity, and function	0-100	Total score calculated using IKDC questionnaire scoring algorithm; transformed to a 0-100 scale	Higher score = better knee function; 0–40 poor, 40–60 fair, 60–80 good, >80 excellent
Foot and Ankle Ability Measure (FAAM)-activities of daily living (ADL) subscale	Activities of daily living	0-100%	(Sum of item scores/Maximum possible score) × 100	Higher score = better foot and ankle function
Foot and Ankle Ability Measure (FAAM)-sports subscale	Sports and high-level functional activities	0-100%	(Sum of item scores/Maximum possible score) × 100	Higher score = better sports-related ankle function

Statistical analysis was performed using SPSS® version 26.0 (IBM Corp., Armonk, New York, USA). Continuous variables were expressed as mean ± standard deviation, and categorical variables as frequencies and percentages. Intergroup comparisons were conducted using independent t-tests, while within-group comparisons were analyzed using paired t-tests. A p-value < 0.05 was considered statistically significant.

## Results

As shown in Table 2, both groups were comparable with respect to baseline characteristics, including gender distribution and the presence of associated meniscal injuries. The majority of patients had no concomitant meniscal tear, with only a small proportion exhibiting medial or lateral meniscal involvement in both groups.

**Table 2:** Demography and baseline characteristics.

Demography and baseline characteristics (n = 60)	Hamstring (n = 30)	Peroneus longus (n = 30)
Mean age	28.6 ± 6.2 years	29.1 ± 5.9 years
Male	24	19
Female	6	11
Medial meniscal tear	6	6
Lateral meniscal tear	2	2
No meniscal tear	22	22

Table 3 explains that graft length and tunnel dimensions were similar in the two groups. However, the peroneus longus tendon autograft demonstrated a significantly greater graft thickness compared to the hamstring tendon autograft, indicating a structurally larger graft diameter in the peroneus longus group.

**Table 3:** Intergroup comparison of graft and tunnel characteristics between hamstring and peroneus longus tendon groups. Independent sample t-test was used for intergroup comparisons.

Variable	Hamstring (mean ± SD)	Peroneus longus (mean ± SD)	Test statistic	p-value
Graft length (cm)	8.00 ± 0.68	8.05 ± 0.40	t = 0.35	0.731
Graft thickness (mm)	8.23 ± 0.52	9.76 ± 0.96	t = 7.12	<0.001
Femoral tunnel length (mm)	34.2 ± 3.5	33.9 ± 3.1	t = 0.28	0.78
Tibial tunnel length (mm)	48.5 ± 4.2	47.9 ± 3.9	t = 0.46	0.64

The comparison of IKDC knee scores at various follow-up intervals in both groups is presented in Table 4. Preoperatively and at one month postoperatively, IKDC scores were comparable between the hamstring and peroneus longus groups. The three-month follow-up score, the hamstring group demonstrated significantly higher IKDC scores, suggesting a relatively faster early functional recovery. By six months, both groups showed substantial improvement, and the difference in IKDC scores between the two graft types was no longer found to be significant.

**Table 4:** Intergroup comparison of IKDC scores at different follow-up intervals. Independent sample t-test was used for intergroup comparison. IKDC: International Knee Documentation Committee.

Time point	Hamstring (mean ± SD)	Peroneus longus (mean ± SD)	Test statistic	p-value
Pre-op	38.6 ± 5.28	38.6 ± 4.98	t = 0.00	1
One month	20.67 ± 2.47	20.97 ± 5.36	t = 0.28	0.78
Three months	58.23 ± 6.52	52.30 ± 6.82	t = 3.48	0.001
Six months	69.03 ± 2.17	68.07 ± 2.56	t = 1.59	0.12

Functional outcome in both groups was assessed using FAAM scores, which are summarized in Table 5. Baseline FAAM scores did not differ significantly between the two groups. A transient reduction in scores was observed at the one-month follow-up in both groups, followed by progressive improvement at subsequent intervals. At three and six months, FAAM scores were comparable between the hamstring and peroneus longus groups, with no statistically significant intergroup differences, indicating similar ankle- and foot-related functional outcomes.

**Table 5:** Intergroup comparison of FAAM scores at different follow-up intervals. Independent sample t-test was used for intergroup comparison. FAAM: Foot and Ankle Ability Measure.

Time point	Hamstring (mean ± SD)	Peroneus longus (mean ± SD)	Test statistic	p-value
Pre-op	48.80 ± 2.78	50.30 ± 4.44	t = 1.56	0.123
One month	38.73 ± 1.91	39.13 ± 2.01	t = 0.78	0.433
Three months	43.23 ± 2.43	43.97 ± 1.73	t = 1.34	0.184
Six months	64.70 ± 4.47	66.63 ± 3.76	t = 1.82	0.075

The temporal changes in IKDC scores within each group are detailed in Table 6. Both graft groups demonstrated statistically significant improvements in IKDC scores across all postoperative intervals. The most pronounced improvement occurred between the first and the third month scores in both groups. While the hamstring group showed greater improvement in the earlier one to three months, the peroneus longus group exhibited sustained functional gain in the later follow-up period at six months.

**Table 6:** Intra-group changes in IKDC scores over time during follow-up. Paired t-test is employed for intra-group comparisons. IKDC: International Knee Documentation Committee.

Interval	Hamstring mean (change ± SD)	t-value	p-value	Peroneus longus mean (change ± SD)	t-value	p-value
Pre-op to one month	17.93 ± 5.31	18.5	<0.001	17.63 ± 4.07	21.1	<0.001
One to three months	37.57 ± 5.82	28.6	<0.001	31.33 ± 5.29	25.4	<0.001
Three to six months	10.80 ± 5.19	11.2	<0.001	15.77 ± 5.54	13.9	<0.001

The changes in FAAM scores are presented in Table 7. Both groups showed significant improvements in FAAM scores in the following months of the surgery. The improvement was gradual during the first one to three months, but a marked increase was observed between three and six months. The magnitude of improvement was comparable between the hamstring and peroneus longus groups.

**Table 7:** Intra-group changes in FAAM scores over time during follow-up. Paired t-test is employed for intra-group comparisons. FAAM: Foot and Ankle Ability Measure.

Interval	Hamstring mean (change ± SD)	t-value	p-value	Peroneus longus mean (change ± SD)	t-value	p-value
Pre-op to one month	10.07 ± 1.87	26.3	<0.001	11.17 ± 2.99	18.4	<0.001
One to three months	4.50 ± 1.14	21.6	<0.001	4.83 ± 1.34	19.2	<0.001
Three to six months	21.47 ± 4.36	27.4	<0.001	22.67 ± 3.51	30.1	<0.001

## Discussion

The determination of an optimal autologous graft for anterior cruciate ligament (ACL) reconstruction remains a subject of persistent scientific scrutiny, particularly in relation to balancing biomechanical adequacy, graft dimensional sufficiency, and donor-site morbidity. Hamstring tendon autografts, despite their widespread adoption, are inherently limited by interindividual variability in graft diameter, potential inadequacy in patients with smaller anthropometric profiles, and concerns regarding postoperative deficits in knee flexor strength and altered neuromuscular function. These limitations have catalyzed investigation into alternative autograft sources, with the peroneus longus tendon emerging as a promising candidate. Recent biomechanical and clinical investigations have demonstrated that the peroneus longus tendon possesses favorable tensile strength and elastic modulus comparable to conventional hamstring grafts, while providing reliable knee joint stability and satisfactory patient-reported functional outcomes following ACL reconstruction. Importantly, accumulating evidence indicates that harvesting the peroneus longus tendon does not result in clinically significant compromise of ankle strength, stability, proprioception, or gait parameters, thereby suggesting a minimal donor-site functional penalty. Collectively, these findings support the peroneus longus tendon autograft as a biomechanically robust and functionally viable alternative for ACL reconstruction, warranting further high-quality comparative trials to delineate its long-term efficacy and safety profile [11-14].

The present study evaluated clinical and functional outcomes following anterior cruciate ligament reconstruction using hamstring tendon and peroneus longus tendon autografts. The present findings suggest that both graft options are associated with comparable short-term functional outcomes, with observed differences in graft morphology and early postoperative recovery demonstrating small effect sizes and limited clinical relevance. Functional scores and objective stability measures did not exceed established minimal clinically important difference thresholds. These results are concordant with previously published comparative studies reporting equivalent postoperative knee stability and functional recovery profiles between the two graft modalities [11-14].

Baseline demographic characteristics and associated meniscal injuries were comparable between the two groups, reducing potential confounding factors and supporting the validity of functional outcome comparisons. The significantly greater graft thickness observed in the peroneus longus group has been consistently reported in earlier studies, which documented that peroneus longus tendons generally yield larger graft diameters compared with hamstring tendons [11-13,16]. Larger graft diameter has been suggested to improve graft strength and reduce failure risk, although the clinical outcome correlations remain inconclusive [17].

Knee-specific outcomes, assessed using IKDC scores, revealed no significant differences between groups in the preoperative and early postoperative periods, except for a transient advantage in the hamstring group at three months. Similar early functional superiority of hamstring grafts has been reported in previous studies, possibly reflecting more familiar rehabilitation protocols and early donor-site adaptation [12,14,20]. However, consistent with multiple comparative reports, this early difference diminished over time, and no statistically significant difference was observed at six months [11,13,15]. These findings support prior conclusions that the peroneus longus tendon autograft is not inferior to the hamstring tendon autograft with respect to knee function.

The potential impact of peroneus longus tendon harvest on ankle function remains a major concern. In the present study, FAAM scores did not differ significantly between groups at any follow-up interval. This finding corroborates multiple studies that reported preserved ankle function following peroneus longus harvest, with no clinically meaningful donor-site morbidity [11,14,21]. Some authors have suggested that compensation by the peroneus brevis muscle and surrounding ankle stabilizers may account for the maintenance of ankle strength and proprioception [20,22].

Within group analysis demonstrated statistically significant improvement in IKDC scores across all postoperative intervals in both graft groups. The greatest improvement occurred between one and three months, corresponding to the transition from early rehabilitation to the strengthening and neuromuscular training phases [12,14,19].Although the hamstring group exhibited greater early improvement, the peroneus longus group showed sustained gains at later follow-up, resulting in equivalent outcomes by six months, consistent with earlier reports [11,13,15].

Similarly, FAAM scores showed significant improvement over time in both groups, with the largest increase occurring between three and six months. Comparable recovery patterns have been reported in previous studies evaluating ankle-related outcomes following peroneus longus tendon harvest, with no long-term functional compromise [14,19,20]. These findings reinforce the functional safety of the peroneus longus tendon autograft in ACL reconstruction.

The present study aligns with prior literature and contributes additional evidence supporting the use of the peroneus longus tendon autograft as a reliable alternative to the hamstring tendon autograft in anterior cruciate ligament reconstruction [11-16]. The consistently observed greater graft thickness, combined with comparable knee and ankle functional outcomes, suggests that the peroneus longus tendon may be particularly advantageous in cases where hamstring graft size is insufficient or preservation of hamstring strength is desirable.

The limitations of the study may also be taken into cognizance, as the sample size was relatively small, which may limit the generalizability of the findings. Secondly, the follow-up duration was only six months due to time constraints of the study, and long-term graft survival and functional outcomes could not be assessed. The objective biomechanical assessment testing, such as isokinetic muscle strength measurement, was not performed. Finally, randomization was performed, but blinding of subjects was not feasible during the surgery, which may introduce potential bias. Further studies with a larger sample size and long-duration follow-up periods are needed to confirm these findings.

## Conclusions

Both hamstring tendon and peroneus longus tendon autografts resulted in satisfactory and comparable short-term clinical and functional outcomes following anterior cruciate ligament reconstruction. Although the hamstring tendon autograft demonstrated marginally superior early postoperative knee function, this advantage was transient, with no statistically significant differences in knee-related outcomes observed at six months. The peroneus longus tendon autograft consistently provided a greater graft diameter and was not associated with clinically meaningful impairment of ankle function. Collectively, these findings support the peroneus longus tendon autograft as a safe and effective alternative to the hamstring tendon autograft, particularly in cases of insufficient hamstring graft size or when preservation of hamstring function is a priority. Larger, adequately powered studies with extended follow-up are required to determine long-term functional outcomes and graft survival.
